# Physiological and Biochemical Adaptations to Repeated Drought–Rehydration Cycles in *Ochroma lagopus* Swartz: Implications for Growth and Stress Resilience

**DOI:** 10.3390/plants14111636

**Published:** 2025-05-27

**Authors:** Yuanxi Liu, Jianli Sun, Cefeng Dai, Guanben Du, Rui Shi, Junwen Wu

**Affiliations:** 1 College of Forestry, Southwest Forestry University, Kunming 650224, China; lyx1997@swfu.edu.cn (Y.L.); 1695136189@swfu.edu.cn (J.S.); daicefeng@swfu.edu.cn (C.D.); 2 College of Materials and Chemical Engineering, Southwest Forestry University, Kunming 650224, China; guanben@swfu.edu.cn; 3 Yunnan Provincial Key Laboratory for Conservation and Utilization of In-Forest Resource, Southwest Forestry University, Kunming 650224, China; shirui@swfu.edu.cn

**Keywords:** *Ochroma lagopus* Swartz, growth, biomass, physiology and biochemistry, non-structural carbohydrates

## Abstract

*Ochroma lagopus* Swartz is a rapidly growing plant known for its lightweight wood; it is widely utilized for timber production and ecological restoration. We investigated the effects of different numbers of drought–rehydration cycles on *O. lagopus* seedlings cultivated at the Xishuangbanna Tropical Botanical Garden of the Chinese Academy of Sciences. The experiment comprised three treatments: normal watering (CK, 80–85% field capacity), one drought–rehydration cycle (D1, one rewatering), and three drought–rehydration cycles (D2, three rewaterings). We characterized the effects of these treatments on seedling growth, biomass allocation, non-structural carbohydrates (NSCs), malondialdehyde (MDA), catalase (CAT) activity, peroxidase (POD) activity, superoxide dismutase (SOD) activity, proline content, and soluble protein content. The number of drought–rehydration cycles had a significant effect on the growth characteristics and physiological and biochemical properties of leaves. As the number of drought–rehydration cycles increased, the height increased significantly (by 17.17% under D2). The leaf biomass ratio, soluble sugar content, and starch content decreased (15.05%, 15.79%, and 46.92% reductions under the D2 treatment); the stem biomass ratio and root biomass ratio increased; CAT activity increased and then decreased (it was highest at 343.67 mg·g^−1^·min^−1^ under D1); and the POD and SOD activities, the MDA content, the soluble protein content, and the soluble sugar/starch ratio increased significantly (395.42%, 461.82%, 74.72%, 191.07%, and 59.79% higher under D2). The plasticity of growth was much greater than that of physiological and biochemical traits. In summary, *O. lagopus* seedlings adapted to multiple drought–rehydration cycles by increasing the accumulation of soluble proteins (likely associated with osmotic protection), activating enzymes (POD and SOD), promoting the conversion of NSCs (increasing stored carbon consumption), and allocating more biomass to plant height growth than to diameter expansion. Under climate change scenarios with intensified drought frequency, elucidating the drought resistance mechanisms of *O. lagopus* is critical to silvicultural practices in tropical plantation.

## 1. Introduction

With the intensification of human activities and global climate change, the frequency, intensity, and duration of drought events are increasing, which affects forest growth and development and poses severe threats to the biodiversity and stability of forest ecosystems [1]. Water is a critical component of forests that is involved in nearly all physiological and biochemical processes in trees, drought directly affects tree growth and development and can even lead to tree mortality [2]. Yunnan Province is particularly prone to seasonal droughts due to its unique geographical location and complex topography [3]. Plants adapt to drought stress by modifying their growth, physiological, and morphological structures to enhance their resilience [4,5]. Drought can also induce the closure of stomata to reduce water loss through transpiration [6]. Additionally, plants may undergo a series of adaptive changes in leaf structure, such as increased leaf and cuticle thickness, to improve water storage capacity [7].

Since plants do not always experience continuous drought conditions, rapid rehydration following rainfall has significant effects on plants. Some plants can modify their physiological functions in response to drought; these plants have developed unique drought-resistant strategies to avoid mortality and exhibit compensatory growth after rehydration, which restores their basal metabolic rates to pre-drought levels [8,9]. Clarifying the responses of different plants to drought stress and subsequent rehydration is critically important for improving plant survival and productivity. Previous studies have found that rehydration after drought can restore physiological functions to some extent, compensating for drought-induced damage, increasing rates of photosynthesis, and accelerating growth [10]. However, the compensatory growth after drought is often limited, and the extent of recovery may depend on the severity and duration of the drought stress experienced prior to rehydration [11,12]. When plants that have previously undergone water stress and rehydration experience drought again, their enhanced drought adaptation may be associated with the expression of genes involved in stress tolerance, osmotic regulation, and antioxidant capacity, which enhances their resistance to drought at the physiological, biochemical, and molecular levels [13,14,15]. However, some studies suggested that previously acclimated plants do not necessarily show improved drought resistance upon subsequent drought exposure [16,17,18]. Therefore, as the frequency of droughts increases due to global climate change, studies of the mechanisms underlying the responses of plants to repeated droughts are needed.

In natural environments, particularly tropical rainforest climates, drought often occurs between periods of rainfall; indeed, repeated drought–rehydration cycles are more common than prolonged drought events. Increases in the concentrations of greenhouse gases have led to increases in the frequency of extreme climate events; plants will be increasingly exposed to drought–rehydration cycles [19]. Therefore, the ability of plants to cope with cyclical droughts and recover after rewatering is critically important [20]. Although potential drought-resistant mechanisms in plants have been extensively explored, nevertheless, plants adapt to cyclical droughts remain unknown [21]. Investigation of the effects of repeated drought cycles and subsequent rewatering on growth characteristics and physiological–biochemical traits is necessary for clarifying the mechanisms by which plants adapt to cyclical droughts.

Under drought stress, plants activate their own physiological response mechanisms, such as mitigating drought adversity by altering the expression of drought-resistant genes, protective enzyme activities, the content of non-structural carbohydrates (NSCs), and the content of osmoregulatory substances [22,23,24]. NSCs are important substances involved in the life processes of forest trees, which have major effects on the growth and development of trees [25]. The main components are soluble sugars (e.g., fructose, glucose, and sucrose) and starch, NSC play a role in the metabolic processes of plants and provide energy substances, which can be transported and interconverted in plants [26]. Soluble sugars play an important role in the drought resistance [27] of plants, and starch, a long-term energy storage substance in plant tissues, is one of the most important forms of carbohydrates present in plant organs; furthermore, the two can be interconverted to increase the stress tolerance [28]. The accumulation of reactive oxygen species (ROS) and MDA under stress induces an increase in osmotic substances, such as soluble sugars, soluble proteins, and proline, as well as an increase in the activities of protective enzymes, such as peroxidase (POD), superoxide dismutase (SOD), and catalase (CAT), to enhance drought tolerance [24]. The indicators of phenotypic plasticity reflect the adaptive capacity of plants in relation to the environment [23]. There is thus a need to clarify changes in the physiological and ecological characteristics as well as their phenotypic plasticity to elucidate the mechanisms by which plants adapt to adverse environments.

*Ochroma. lagopus* is a perennial tropical fast-growing plant native to low-latitude regions near the equator. It was introduced into Yunnan and Hainan Province, China, in the 1960s. *O. lagopus* is the world’s lightest commercial wood, and it is widely used for wind power generation and in lightweight sandwich panels; it is also used for high-speed rail car manufacturing and drone production. Most research has focused on the cultivation of *O. lagopus* due to the relatively recent increase in *O. lagopus* cultivation in China. The Xishuangbanna region of Yunnan province experiences seasonal droughts, which affect local agriculture, ecosystems, and communities. Climate change, the precipitation distribution, topography, and human activities contribute to the frequent occurrence of drought in this area. The results of this study clarify the effects of repeated drought stress and rewatering on the growth and development of *O. lagopus* seedlings and enhance our understanding of the drought adaptability in Xishuangbanna area.

Most recent studies of *O. lagopus* have focused on the early stages of seed germination [29]. Given that *O. lagopus* has been increasingly applied in various fields in China and that its cultivation in tropical regions is increasing under changing global climate conditions, there is a need to refine cultivation techniques for *O. lagopus*. O. lagopus, despite its ecological and economic importance, presents a paradox: its rapid growth and large leaf area enhance carbon fixation but may also increase vulnerability to recurrent droughts due to high transpiration demand and low wood density. This knowledge gap hinders the development of science-based strategies for its sustainable plantation management under fluctuating water regimes. Here, we analyzed the effects of repeated drought–rehydration cycles on the growth, biomass allocation, leaf enzyme activities, and osmotic regulatory substances of *O. lagopus* seedlings. Our goal was to shed light on the adaptation and growth strategies of *O. lagopus* seedlings under alternating rainfall and drought conditions. Our findings have implications for *O. lagopus plantation* management. Our study examined (1) whether growth indices and the biomass allocation of *O. lagopus* seedlings change as the number of drought–rehydration cycles increases; (2) whether the NSC content, osmoregulatory substances, and antioxidant enzyme activity products of *O. lagopus* leaves change as the number of drought–rehydration cycles increase; and (3) whether there are differences in the plasticity of growth trait indices and physiological trait indices in response to increases in the number of drought–rehydration cycles and the correlations between them. The effects of the NSC content, osmoregulatory mechanisms, and antioxidant enzyme activity on the growth of *O. lagopus* seedlings are also discussed.

## 2. Results

### 2.1. Phenotypic Changes in O. lagopus Seedlings as the Number of Drought–Rehydration Cycles Increases

As the number of drought–rehydration cycles increased, the height of *O. lagopus* seedlings increased significantly and the ground diameter decreased (Table 1). The number of drought–rehydration cycles significantly affected the height of *O. lagopus* seedlings (*F* = 10.048, *p* < 0.05) (Table 2). Seedling height was 1.39% and 17.17% higher in the D1 and D2 treatments than in the CK treatment, respectively, and the increase in height in the D2 treatment was significant (*p* < 0.05). The ground diameter was 1.14% and 18.16% lower in the D1 and D2 treatments, respectively, and the decrease in ground diameter in the D2 treatment was significant.

### 2.2. Effects of Increases in the Number of Drought–Rehydration Cycles on Biomass Changes and the Distribution of O. lagopus Seedlings

**Figure 1:**
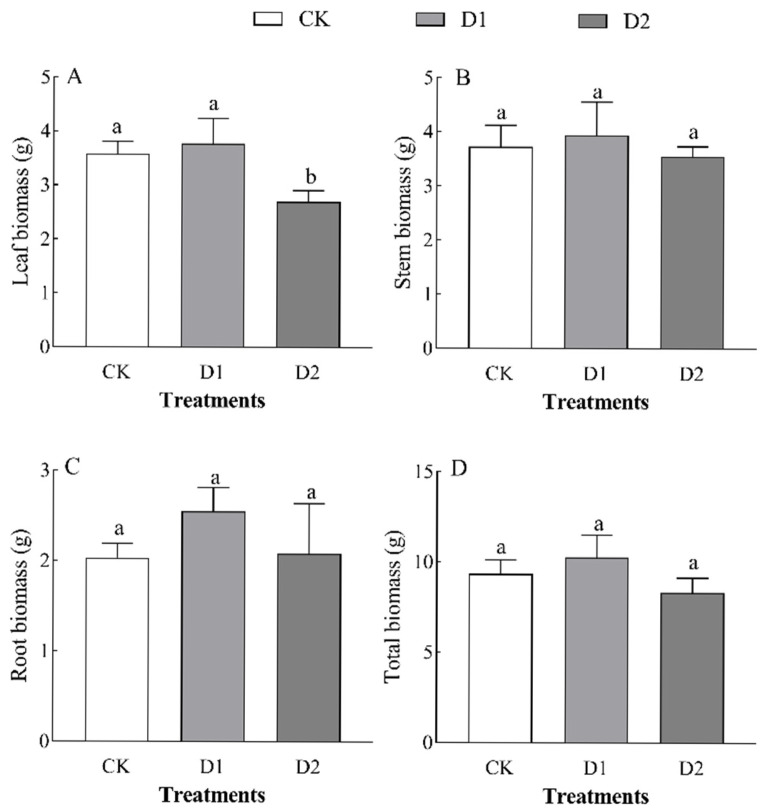
Effect of increases in the number of drought–rehydration cycles on *O. lagopus* biomass. (**A**): Leaf biomass; (**B**): Stem biomass; (**C**): Root biomass; (**D**): Total biomass; CK: normal watering; D1: one drought–rehydration cycle; D2: three drought–rehydration cycles. The error bars indicate the standard deviations of the means (n = 4). Different letters indicate significant differences between treatments (*p* < 0.05).

**Figure 2:**
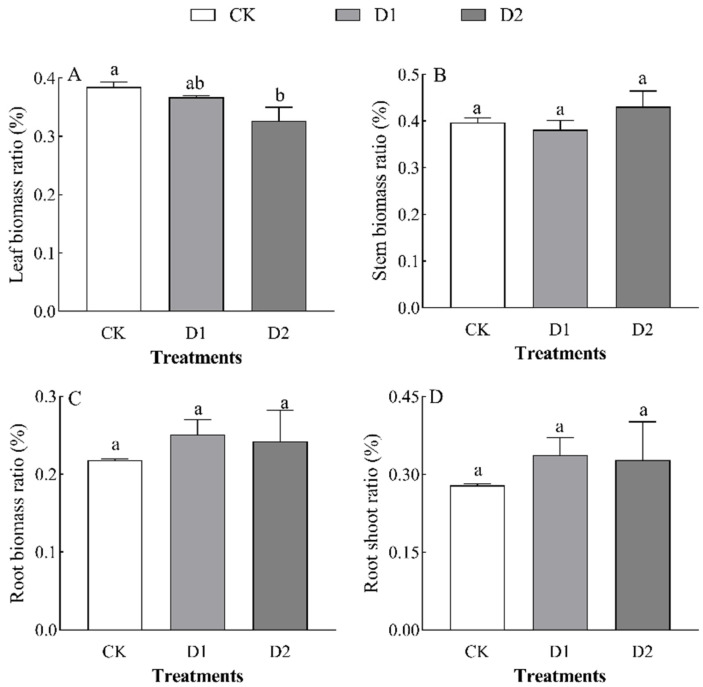
Effect of increases in the number of drought–rehydration cycles on the biomass allocation of *O. lagopus.* (**A**): Leaf biomass ratio; (**B**): Stem biomass ratio; (**C**): Root biomass ratio; (**D**): Root shoot ratio; CK: normal watering; D1: one drought–rehydration cycle; D2: three drought–rehydration cycles. The error bars indicate the standard deviations of the means (n = 4). Different letters indicate significant differences between treatments (*p* < 0.05).

Increases in the number of drought–rehydration cycles had no significant effect on the biomass of each organ in *O. lagopus* seedlings (Table 2). The leaf biomass, stem biomass, root biomass, and total biomass of *O. lagopus* seedlings increased and then decreased as the number of drought–rehydration cycles increased, and the highest values of these variables were observed in the D1 treatment (Figure 1). Leaf biomass was lowest in the D2 treatment (2.70 g), and it was 24.43% lower in the D2 treatment than in the control (*p* < 0.05); stem biomass, root biomass, and total biomass did not significantly differ (*p* > 0.05) among treatments. Leaf biomass was 4.55% and 15.05% lower in the D1 and D2 than in the CK, respectively, and the stem biomass ratio, root biomass ratio, and root/crown ratio increased with the number of drought–rehydration cycles (Figure 2B–D); however, no significant differences were observed among the treatments.

### 2.3. Changes in the NSCs of O. lagopus Leaves as the Number of Drought–Rehydration Cycles Increases

**Figure 3:**
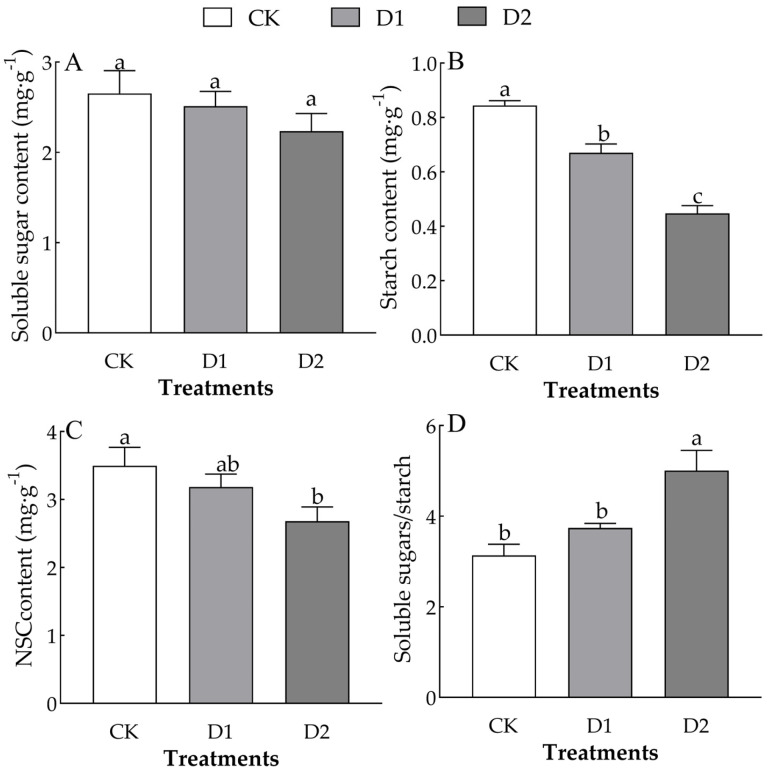
Effect of increases in the number of drought–rehydration cycles on the NSCs of *O. lagopus* leaves. (**A**): Soluble sugar content; (**B**): Starch content; (**C**): NSCs content; (**D**): Soluble sugar/ Starch; CK: normal watering; D1: one drought–rehydration cycle; D2: three drought–rehydration cycles. The error bars indicate the standard deviations of the means (n = 4). Different letters indicate significant differences between treatments (*p* < 0.05).

Different numbers of drought–rehydration cycles only significantly affected the starch content and the soluble sugar/starch ratio in the leaves of *O. lagopus* seedlings (*F* = 55.494, *p* < 0.01; *F* = 10.270, *p* < 0.05) (Table 2). The leaf soluble sugar, starch, and NSC content of *O. lagopus* seedlings decreased as the number of drought–rehydration cycles increased. The soluble sugar content was 5.31% and 15.79% lower in the D1 and D2 than in the CK, respectively (Figure 3A). The starch content was 20.50% and 46.92% lower in the D1 and D2 than in the CK, respectively (Figure 3B). The NSC content was 8.98% and 23.30% lower in the D1 and D2 than in the CK, respectively (Figure 3C). The soluble sugar/starch ratio was 19.32% and 59.79% higher in the D1 and D2than in the CK, respectively (Figure 3D).

### 2.4. Changes in the MDA Content and Peroxidase Enzyme Activity of O. lagopus Needles as the Number of Drought–Rehydration Cycles Increases

**Figure 4:**
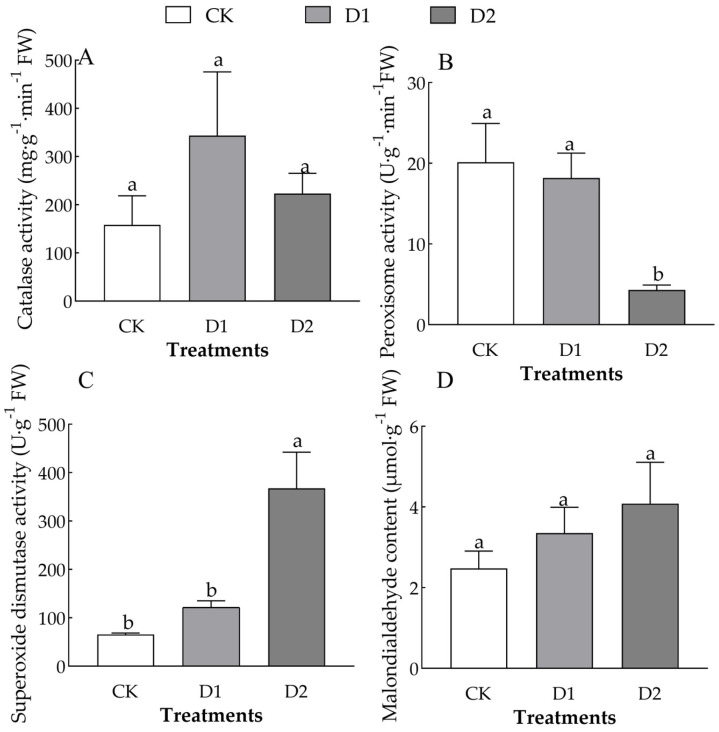
Effect of increases in the number of drought–rehydration cycles on the POD activity and MDA content of *O. lagopus* leaves. (**A**): CAT; (**B**): POD; (**C**): SOD; (**D**): MDA; CK: normal watering; D1: one drought–rehydration cycle; D2: three drought–rehydration cycles. The error bars indicate the standard deviations of the means (n = 4). Different letters indicate significant differences between treatments (*p* < 0.05).

The number of drought–rehydration cycles had a significant effect on POD and SOD activity in the leaves of *O. lagopus* seedlings (*F* = 6.756, *p* < 0.05; *F* = 13.475, *p* < 0.01) (Table 2). As the number of drought–rehydration cycles increased, leaf CAT activity increased and then decreased (Figure 4A), and the SOD activity and MDA content gradually increased (Figure 4B–D). CAT activity was 117.61% and 41.22% higher in the D1 and D2, respectively, than in the CK. POD activity was 321.23% and 366.40% lower in the D1 and D2, respectively, than in the CK. SOD activity was 85.62% and 562.82% higher in the D1 and D2 than in the CK, respectively. The MDA content was 35.28% and 64.72% higher in the D1 and D2 than in the CK, respectively.

### 2.5. Effect of Increases in the Number of Drought–Rehydration Cycles on the Proline and Soluble Protein Content of O. lagopus Leaves

**Figure 5:**
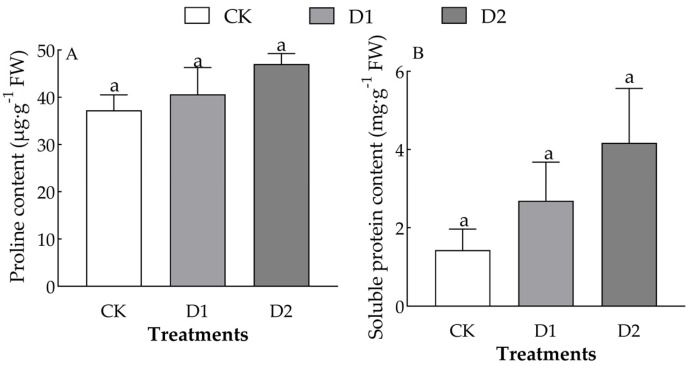
Effects of increases in the number of drought–rehydration cycles on the proline and soluble protein content of *O. lagopus* leaves. (**A**): Proline; (**B**): Soluble protein; CK: normal watering; D1: one drought–rehydration cycle; D2: three drought–rehydration cycles. The error bars indicate the standard deviations of the means (n = 4). Different letters indicate significant differences between treatments (*p* < 0.05).

Increases in the number of drought–rehydration cycles only significantly affected the content of proline and soluble protein in the leaves of *O. lagopus* seedlings (*F* = 2.746, *p* = 0.142; *F* = 1.769, *p* = 0.249) (Table 2). The leaf proline and soluble protein content of *O. lagopus* seedlings increased with the number of drought–rehydration cycles (Figure 5). The leaf free proline content was 9.02% and 42.74% higher and the soluble protein content was 74.44% and 172.04% higher in the D1 and D2, respectively, than in the CK.

### 2.6. Plasticity Analysis of the Effects of Increases in the Number of Drought–Rehydration Cycles on the Growth and Leaf Physiological and Biochemical Indexes of O. lagopus Seedlings

**Figure 6:**
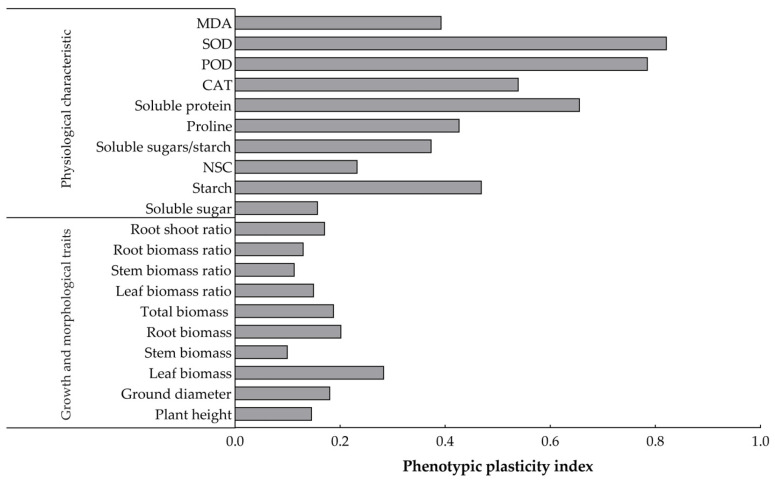
Phenotypic plasticity indices of growth and physiological and biochemical traits of *O. lagopus* seedlings as the number of drought–rehydration cycles increases.

The plasticity values of all the trait indexes of *O. lagopus* seedlings that experienced different numbers of drought–rehydration cycles ranged from 0.100 to 0.822 (Figure 6). The plasticity indexes of the growth trait indexes were small, and the plasticity indexes of the physiological trait indexes were relatively large. The largest plasticity index for the growth trait index was for leaf biomass (0.284), and the smallest was for stem biomass (0.100). Starch (0.470) had the largest plasticity index for NSCs and SOD (0.822), followed by POD (0.786) for leaf enzyme activities. The plasticity index of soluble protein (0.656) was highest among the osmoregulatory substances.

### 2.7. Correlations Between Growth Indices and Leaf Physiological and Biochemical Indices of O. lagopus Seedlings as the Number of Drought–Rehydration Cycles Increases

**Figure 7:**
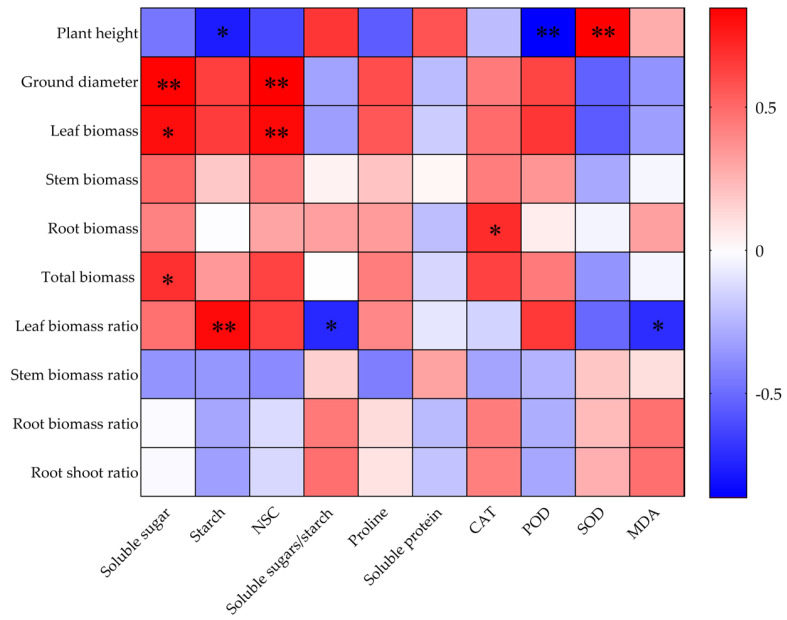
Correlations of growth traits with leaf physiological and biochemical trait indices in *O. lagopus* seedlings as the number of drought–rehydration cycles increases. * *p* < 0.05; ** *p* < 0.01.

Correlations of the growth indices with leaf physiological and biochemical indices of *O. lagopus* seedlings are shown in Figure 7. The leaf soluble sugar content was significantly and positively correlated with ground stem biomass, leaf biomass, and total biomass. The starch content was significantly positively correlated with leaf biomass and negatively correlated with plant height, and the NSC content was highly significantly positively correlated with ground stem and leaf biomass. The soluble sugar/starch ratio was significantly and negatively correlated with the leaf biomass ratio. CAT was significantly and positively correlated with root biomass. POD was significantly and negatively correlated with plant height. SOD was significantly and positively correlated with plant height. MDA was significantly and negatively correlated with the leaf biomass ratio.

## 3. Discussion

### 3.1. Effects of Increases in the Number of Drought–Rehydration Cycles on the Growth and Biomass of O. lagopus Seedlings

The adaptation of plants to water-deficit conditions is a complex process influenced by various external environmental conditions and intrinsic drought tolerance mechanisms [30]. Under drought stress, the early morphological responses of plants are common, such as reductions in height, ground diameter, and total dry biomass [30,31]. Generally, drought environments hinder normal physiological activities and suppress plant growth. To ensure survival, plants typically reduce their growth rates to conserve water [30]. In this study, the height of *O. lagopus* seedlings was 1.39% and 17.17% higher in the D1 and D2 than in the CK, respectively; the ground diameter was 1.14% and 18.16% lower in the D1 and D2 than in the CK, respectively (Table 1). This indicates that *O. lagopus* seedlings are not highly sensitive to a single drought–rehydration cycle but respond more strongly to subsequent drought–rehydration cycles. The responses of height and ground diameter to drought–rehydration cycles differed. The leaf biomass and its allocation ratio decreased under drought–rehydration cycles (Figure 1A and Figure 2A). Conversely, initial increases followed by decreases were observed for other biomass categories; the stem biomass ratio, root biomass ratio, and root-to-shoot ratio all increased (Figure 2). This suggests that after repeated drought–rehydration cycles, resource allocation to vital organs is prioritized in *O. lagopus*, which increases the photosynthetic capacity of the leaves and the nutrient absorption capacity of the roots. The reduction in leaf biomass likely stems from the adaptive strategies of *O. lagopus*, such as reducing leaf area, increasing leaf thickness, and enhancing cuticle thickness to improve the water storage capacity [7] and reduce transpiration under extreme conditions [32].

Despite the supplementary water provided during rewatering, the damage caused by drought is often cumulative, which leads to an overall reduction in biomass. *O. lagopus* modifies biomass allocation among different organs during multiple drought–rehydration cycles [33]. Following drought, plants may prioritize root growth to access water, and rewatering and improved moisture conditions may promote the aboveground growth of *O. lagopus* to promote resource acquisition. This is consistent with the results of previous research showing that drought–rehydration results in a more balanced root-to-shoot ratio, which enhances the allocation of dry matter to roots to improve water utilization under drought stress and promotes adaptation to environmental stress [34]. This also supports the optimal allocation theory, which posits that resources should be preferentially allocated to the most limiting organs following stress exposure [35]. However, this finding contrasts with the results of Deng et al. [36] showing that drought stress did not affect the root biomass allocation of *Pinus massoniana* Lamb., which likely stemmed from differences in growth environments between *O. lagopus* and *P. massoniana*, with *O. lagopus* being a tropical species.

### 3.2. Effects of Different Numbers of Drought–Rehydration Cycles on Leaf Physiology and Biochemistry of O. lagopus Leaves

Under stress, plants accumulate a substantial amount of ROS, and antioxidant enzymes such as SOD, POD, and CAT play crucial roles in scavenging free radicals. High enzyme activity can enhance plant stress resistance [22]. Antioxidant enzymes are vital for protecting cells against ROS damage. When plants experience abiotic stress, they often modulate the activities of enzymes to mitigate internal damage [37]. ROS can damage cellular membrane systems, and when ROS levels exceed a certain threshold, membrane lipid peroxidation occurs, which leads to the accumulation of malondialdehyde (MDA) [15]. Therefore, the MDA content not only serves as an indicator of plant damage but also as a marker of plant senescence [18,38].

As the number of drought–rehydration cycles increased, the CAT activity in the leaves of *O. lagopus* seedlings initially increased and then decreased, and it remained higher in the drought–rehydration treatments than in the CK (Figure 4A). POD activity, SOD activity, and MDA content increased gradually (Figure 4B–D). This indicates that CAT activity in *O. lagopus* seedlings is particularly sensitive to drought, although its activity decreased after three drought cycles. These changes reflect a continuous recovery process from oxidative damage; the increase in SOD and POD may serve as a compensatory mechanism to mitigate ROS accumulation. The cumulative effects of repeated drought–rehydration cycles might result in sustained ROS production, exceeding the scavenging capacity of antioxidant enzymes; this can cause the antioxidant system to be overburdened and reduce enzyme activity [18]. The activity of antioxidant enzymes was enhanced in *O. lagopus*, suggesting that the increased activity of plant antioxidant enzymes is associated with short-term drought stress memory [39,40]. This up-regulation of the plant’s defense response significantly enhances the drought tolerance of plants [41,42]. Moreover, SOD and POD activities were significantly increased, suggesting that repeated drought–rehydration cycles effectively promoted SOD and POD activities, which rapidly alleviated the stress associated with the critical period of re-drought [18]. This indicates that SOD and POD activities are critically important for regulating the drought tolerance of *O. lagopus* seedlings. These findings indicate that *O. lagopus* seedlings may retain a “stress imprint” after repeated drought–rehydration treatments, which modulates their physiological responses to subsequent drought events.

NSCs are key intermediates between carbon assimilation (photosynthesis) and growth utilization (carbon consumption), and their storage levels reflect the balance between carbon uptake and expenditure [43]. Changes in the NSC content can provide insights into plant growth status and drought adaptation [23]. In our study, the soluble sugar, starch, and NSC content in the leaves of *O. lagopus* seedlings decreased, and the soluble sugar/starch ratio increased as the number of drought–rehydration cycles increased (Figure 3). This stems from the high consumption of soluble sugars during drought–rehydration cycles, as these sugars stabilize cell membranes and protect enzymes and proteins from oxidative damage. As the number of drought–rehydration cycles increases, this consumption of sugars may continue, leading to a gradual decrease in the soluble sugar content. Moreover, drought conditions typically reduce photosynthetic efficiency, thus decreasing sugar synthesis [9,44]. Additionally, drought can affect the transport and distribution of sugars within the plant, further lowering the soluble sugar content in leaves [45].

The decrease in the starch content stems from the accelerated breakdown of starch to obtain energy and carbon sources under stress conditions. Under drought conditions, plants convert starch into soluble sugars for use. As the number of drought–rehydration cycles increases, starch degradation continues, which results in a gradual decline in the starch content [9,44]. Drought conditions may obstruct starch synthesis pathways [46]. The increase in the soluble sugar/starch ratio stems from the relatively slower consumption rate of soluble sugars compared with starch degradation under drought stress. Soluble sugars perform various critical physiological functions in plants, and starch primarily serves as an energy reserve. Therefore, starch degradation may be more rapid and complete during drought–rehydration cycles, leading to a higher soluble sugar/starch ratio [45]. Although rewatering may restore photosynthesis and sugar synthesis to some extent, the recovery rate of soluble sugars is likely faster than that of starch. This is because soluble sugars can be synthesized and transported more rapidly than starches, as the synthesis of starch requires more time and energy. Under repeated drought conditions, the seedlings likely developed enhanced metabolic turnover (indicating partial drought acclimation) and sustained higher cellular turgor pressure (thereby improving apoplastic water uptake capacity).

Osmotic regulation is a crucial mechanism by which plants resist adverse stress conditions. An increase in osmotic substances helps regulate the osmotic potential and maintain the osmotic balance, thereby enhancing the adaptability of plants to stress [47]. In this study, the proline content in the leaves of *O. lagopus* seedlings increased gradually with the number of drought–rehydration cycles, and the soluble protein content was significantly higher in the D2 than in the CK (Figure 5). This suggests that the accumulation of proline and soluble protein metabolites may also enhance the resistance of plants to drought stress [48]. The content of soluble proteins, which are an important storage form of amino acids [49], gradually increased after the number of repeated drought–rehydration cycles increased, and this enhanced the stability of cell structure and function by promoting protein synthesis to meet the needs of *O. lagopus* growth. Under repeated drought stress, plants may prioritize rapid-response protein repair mechanisms (e.g., antioxidant enzyme activation) over long-term accumulation of osmotic solutes. It is also possible that this is related to the transient recovery and stress memory of plants after rewatering. For example, in some plants, proline degradation is inhibited during water stress, and re-watering triggers the opposite regulatory pathway (i.e., the activation of proline synthesis) [50].

### 3.3. Plasticity Analysis of the Growth and Physiological and Biochemical Indices of O. lagopus Seedlings in Response to Different Numbers of Drought–Rehydration Cycles

Investigation of the growth, physiological, and biochemical responses of plants under repeated drought–rehydration cycles provides insights into how plants perceive environmental changes and make adjustments to maintain their survival and reproduction. Our study revealed that plasticity ranges from 0.100 to 0.822 (Figure 6), which highlights the high adaptability and regulatory capacity of *O. lagopus* seedlings in response to fluctuations in water availability. Different plastic responses were observed between growth traits and physiological–biochemical traits. Although the overall plasticity index was relatively low for growth traits, noticeable variation was still observed, including the higher plasticity index for leaf biomass. This suggests that *O. lagopus* seedlings may optimize resource allocation by adjusting leaf growth strategies during drought–rehydration cycles, thereby enhancing their survival capacity. In contrast, physiological traits exhibit more pronounced plasticity, which reflects ecological adaptation mechanisms in response to drought and rewatering challenges. Specifically, the high plasticity index of leaf NSCs indicates that *O. lagopus* seedlings can flexibly adjust their internal energy reserves to meet energy demands during drought or rapid growth after rewatering. The plasticity of enzyme activities, such as SOD and POD, was also notable. The significant plasticity of these enzymes reflects the efficient regulation of the antioxidant defense system in *O. lagopus* seedlings, which is crucial for mitigating oxidative stress induced by drought. Additionally, the high plasticity index of soluble proteins among osmotic regulators further confirms that *O. lagopus* seedlings can maintain their water balance and cellular stability through adjustments of cellular osmotic pressure. Under repeated drought–rehydration cycles, complex and strong correlations were observed among growth indicators and physiological–biochemical indicators of *O. lagopus* seedlings. These physiological adaptation mechanisms collectively constitute the survival strategy of *O. lagopus* seedlings in drought–rehydration environments, which demonstrates their marked ecological adaptability and resilience. The positive correlations of the leaf soluble sugar content with stem and leaf biomass and total biomass suggest that soluble sugars are not only a significant energy source but also act as osmotic regulators under drought conditions, which helps maintain the water balance and promotes growth. The negative correlation between starch content and plant height suggests that *O. lagopus* seedlings tend to catabolize limited resources to increase their osmoregulatory capacity and to increase stem height, thereby optimizing resource allocation and improving seedling drought tolerance.

The relationship between the soluble sugar/starch ratio and leaf biomass ratio indicates that *O. lagopus* seedlings exhibit flexibility in carbon allocation. As the number of drought–rehydration cycles increases, seedlings may adjust the ratio of soluble sugars to starch to balance their energy and growth demands, which ensures that they continue to grow under adverse conditions. Changes in the activities of CAT, POD, and SOD reflect the response of *O. lagopus* seedlings to drought stress. The positive correlation between CAT activity and root biomass suggests that enhancing antioxidant enzyme activity helps protect the roots from oxidative damage and maintain normal root function and absorption capacity, which is crucial for plant growth. The negative correlation between POD activity and plant height may reflect the plant’s strategy to reduce height to minimize water evaporation and energy expenditure under drought conditions. Conversely, the positive correlation between SOD activity and plant height suggests that SOD plays an important role in maintaining cell structure and function, which supports normal growth. This strategy helps maintain leaf health and enhance photosynthetic efficiency, which provides necessary energy and material support for plant growth.

## 4. Materials and Methods

### 4.1. Study Area

The experimental site was located in Tree Garden of Southwest Forestry University, Kunming City, Yunnan Province (25°03′ N, 102°46′ E), at an altitude of 1964 m, with an average annual temperature of 16.5 °C, an average annual precipitation of 1035 mm, and a subtropical plateau monsoon climate with a short frost period and distinct wet and dry seasons. The temperature inside the shelter ranges from 18.5 to 37 °C, and the relative humidity of the air ranges from 22.3% to 48.0%.

### 4.2. Plant Material and Experimental Design

All the test materials were selected from *O. lagopus* seedlings sown on 18 February 2023 at the Economic Extension Station of the Xishuangbanna Tropical Botanical Garden, Chinese Academy of Sciences, and transported on 12 April 2023 to the tree garden of Southwest Forestry University. After cultivation, 120 *O. lagopus* seedlings (growth indicator, Table 1) with uniform growth were selected on 14 May and transplanted into 9.25 L plastic pots with trays placed at the bottom. The soil used for potting was a mixture of local red loam soil and humus at a ratio of 3:1 by volume, with a field water-holding capacity of 23.02% by the ring knife method, a bulk density of 1.14 g∙cm^−3^, and a soil pH of 5.9 [23,51]. Weeding and regular water management were carried out to maintain the normal growth of the seedlings.

The seedlings were placed at the experimental site, covered with mulch to prevent underground moisture from affecting the potted plants, and a transparent shelter was built to ensure adequate ventilation and permit the entry of light. The rain shelter was made of transparent material (10 m (L) × 3 m (W) × 3 m (H), which can shelter plants from rain without affecting the entry of light and ventilation. This trial began on 1 July 2023. The first group (CK) was supplied with normal water throughout the experiment, and the relative soil moisture content was maintained at 80–85% field water-holding capacity (FC) (i.e., the actual water content ranged from 36.77% to 41.67%). The second group (D1) experienced only one drought–rehydration treatment, and the soil moisture content was maintained at 80–85% FC for the first 20 days; the watering was then stopped so that the soil moisture content decreased to approximately 35 ± 5% FC (i.e., the actual water content ranged from 14.71% to 19.61%). Cultivation continued for 7 days at this soil water content, and rehydration was carried out until the soil water content reached 80–85% FC, which was maintained until all the experiments were completed. The third group (D2) was subjected to three drought–rehydration treatments, during which there was a drought recovery period, and they were incubated for 2 days at an 80–85% FC soil water content at the end of each rehydration period. After that, the soil water content was allowed to decrease naturally to approximately 35 ± 5% FC (it took approximately 3 days for the soil water content to decrease to 35 ± 5% FC each time). This soil water content was maintained for 7 days, and this cycle was repeated three times (each drought–rehydration cycle lasted approximately 12 days), and samples were taken the next day after the last rehydration period. The experiment lasted a total of 36 days. Actual soil water concentrations were measured using a soil moisture meter (LM61-NC8X318; Guangzhou Huaxi Science and Technology Co., Ltd., Guangzhou, China) and controlled using the weighing method; all pots were weighed daily at 17:00. The soil water content (SWC) was obtained using the weighing method, and the plant soil water content (PSWC) was calculated as the ratio of the soil water content to the soil water-holding capacity in the field; water control or watering was based on the target weight [23,51].

### 4.3. Plant Sampling

At the end of the experiment, the plant height and ground diameter of the *O. lagopus* seedlings were determined using a straight-edge ruler and vernier calipers, respectively. After height and diameter measurements, the seedlings were dug out and rinsed; four representative seedlings were randomly selected from each treatment, rinsed with water, and wiped with absorbent paper to remove the surface moisture (avoiding damaging the root system in the process); they were then brought back to the laboratory for processing. The leaves, stems, and roots were separated and labeled, placed into numbered and labeled envelopes in an oven to kill the tissue for half an hour at 120 °C, and then dried at 80 °C. The dry weight of each part was then determined. The data were recorded, and the leaf biomass ratio (leaf weight/total plant weight), stem biomass ratio (stem weight/total plant weight), root biomass ratio (root weight/total plant weight), and root/shoot ratio ((root weight)/(leaf weight + stem weight)) were calculated for each treatment. The dried samples were ground, sieved, and preserved to determine the content of NSCs in each organ.

### 4.4. Determination of Leaf Physiological and Biochemical Indexes

In this study, NSCs refers to the sum of soluble sugars and starch. Samples (0.05 g) were ground and mixed with 10 mL of distilled water; the mixture was then centrifuged at 4000 r·min^−1^ for 10 min after 10 min in a boiling water bath. The supernatant was then subjected to the anthrone method using a UV-Vis spectrophotometer (HD-UV90; Shandong Holder Electronic Technology Co., Ltd., Tai’an, China). The absorbance value of soluble sugars at 625 nm was determined, and their content was calculated; the starch content in the samples was determined using the precipitates [52].

Malonaldehyde (MDA, μmol·g^−1^ FW): Fresh leaf tissue (0.3 g) was washed, dried, and homogenized in 5 mL 5 % (*w*/*v*) trichloroacetic acid (TCA) under ice-bath conditions. After centrifugation (8000 r/min, 20 min), the supernatant was mixed 1:1 (*v*/*v*) with 0.67 % thiobarbituric acid (TBA), boiled (100 °C, 30 min), and immediately cooled on ice. Following centrifugation at 8000 r/min for 20 min, the absorbance of the mixture was measured spectrophotometrically at 450, 532, and 600 nm [53].

Activities of antioxidant enzymes: Fresh leaf tissue (0.3 g) was homogenized by ice bath in 5 mL pre-cooled 50 mmol L^−1^ phosphate buffer (PBS, pH 7.8, containing 1 % PVP). The homogenate was adjusted to 5 mL with PBS and centrifuged at 10,000 r/min for 30 min at 4 °C. The supernatant was assayed for superoxide dismutase (SOD, U·g^−1^ FW), peroxidase (POD, U·g^−1^·min^−1^ FW), and catalase (CAT, mg^−1^·g^−1^·min^−1^). All procedures were maintained at 0–4 °C. Superoxide dismutase activity was determined by measuring the inhibition of nitroblue tetrazolium photoreduction at 560 nm [54]. Peroxidase activity was determined using the guaiacol method, which involves measuring the change in absorbance at 470 nm to quantify guaiacol oxidation within 3 min [55]. Catalase activity was determined following the procedure of Lei et al. [56] by monitoring the decomposition of H_2_O_2_ through absorbance changes at 240 nm for 3 min.

Proline (μg·g^−1^ FW) and soluble protein (mg·g^−1^ FW): Fresh leaf tissue (0.3 g) was homogenized in 3% (*w*/*v*) sulfosalicylic acid. The homogenate was adjusted to a final volume of 5 mL and centrifuged at 10,000 r/min for 20 min at 4 °C. The supernatant (2 mL) was mixed with equal volumes of glacial acetic acid and acidic ninhydrin reagent, followed by incubation in a boiling water bath for 30 min. After the mixture was cooled, Pro was extracted with toluene as the solvent. Absorbance was measured at 520 nm, and the concentration was calculated using a standard curve [53]. Fresh leaf tissue (0.3 g) was homogenized in 5 mL 50 mM PBS (pH 7.8), and the homogenate was centrifuged at 10,000 r/min for 20 min at 4 °C. The supernatant was mixed with 5 mL Coomassie Brilliant Blue G-250 reagent (Thermo Fisher Scientific, Waltham, MA, USA) and incubated for 5 min. Absorbance was measured at 595 nm and compared with the curve of known concentrations of bovine serum albumin [56].

### 4.5. Statistical Analysis

The normality and homogeneity of the data were assessed (Kolmogorov–Smirnov test) before subsequent statistical analysis. Multiple comparisons were made between the means of the data using Duncan’s test. Correlations between *O. lagopus* growth indicators and leaf physiological indicators were investigated using Pearson correlation analysis. All statistical analyses were performed using SPSS 20.0 (IBM SPSS Statistics, Armonk, NY, USA), and the level of statistical significance was set at *p* = 0.05. Graph Pad Prism 8 was used to make graphs.

Root/shoot ratio = (Leaf biomass + Stem biomass)/(Leaf biomass + Stem biomass + Root biomass).

Plasticity index: P = (X_max_ − X_min_)/X_max_, where X_max_ and X_min_ denote the maximum and minimum values of each indicator, respectively.

## 5. Conclusions

Drought–rehydration cycles significantly affect growth, biomass, biomass partitioning, leaf NSCs, leaf enzyme activities, and osmoregulatory substances in *O. lagopus* seedlings. The increase in the MDA content as the number of drought–rehydration cycles increased indicated that the plants experienced damage that resulted in the inhibition of CAT synthesis or activity, although the activity of antioxidant enzymes was maintained. Increases in POD and SOD were key to mitigating oxidative stress. Correlation and phenotypic plasticity index analyses revealed significant relationships between growth metrics and physiological parameters. *O. lagopus* seedlings tended to allocate limited resources to stem growth to improve aboveground resource acquisition, which led to adjustments in carbon allocation, osmotic regulation (mainly soluble proteins), and enzyme activities (POD and SOD). These findings suggest that *O. lagopus* seedlings improve drought tolerance and resilience by adjusting NSC allocation strategies, regulating enzyme activities, and employing osmoregulatory mechanisms. Although multiple drought–rehydration cycles can disrupt plant growth, *O. lagopus* optimizes growth patterns and biomass allocation and adjusts its leaf NSC storage and catabolism as well as enzyme activities to maintain growth and physiological functions under extreme environmental conditions. Based on the current findings, we recommend implementing hydration regimens for *O. lagopus* seedlings guided by SOD activity increments and starch degradation rates, while adjusting nitrogen fertilization based on growth dynamics and proline accumulation patterns. Future studies should prioritize long-term monitoring of molecular regulatory mechanisms underlying plant adaptation to drought–rehydration cycle frequencies, particularly epigenetic modifications and stress memory-related gene networks.

## Figures and Tables

**Table 1:** Effects of increases in the number of drought–rehydration cycles on the plant height and ground diameter of *O. lagopus* seedings.

Treatments	Plant Height/cm			Ground Diameter/mm		
	Before Treatment	After Treatment	Increment	Before Treatment	After Treatment	Increment
CK	23.32 ± 0.46 a	57.67 ± 2.24 b	34.37 ± 1.83 b	6.93 ± 0.09 a	11.40 ± 1.31 a	4.47 ± 1.39 a
D1	23.14 ± 0.12 a	58.47 ± 0.44 b	35.37 ± 0.33 b	6.43 ± 0.12 a	11.27 ± 0.91 a	4.83 ± 0.79 a
D2	22.91 ± 0.50 a	67.57 ± 1.96 a	44.67 ± 1.50 a	6.46 ± 0.09 a	9.33 ± 0.07 a	3.11 ± 0.03 a

CK: normal watering; D1: one drought–rehydration cycle; D2: three drought–rehydration cycles. Different lowercase letters indicate differences between treatment groups (*p* < 0.05).

**Table 2:** One-way ANOVA of growth attributes and leaf physiological and biochemical indices of *O. lagopus* seedlings as the number of drought–rehydration cycles increases.

Indicators	*F*	*p*
Plant height (cm)	10.048	0.012
Ground diameter (mm)	1.587	0.280
Leaf biomass (g)	3.116	0.118
Stem biomass (g)	0.211	0.815
Root biomass (g)	0.603	0.577
Total biomass (g)	1.019	0.416
Leaf biomass ratio (%)	4.373	0.067
Stem biomass ratio (%)	1.162	0.374
Root biomass ratio (%)	0.44	0.663
Root/shoot ratio (%)	0.438	0.664
Soluble sugars (mg·g^−1^)	1.06	0.404
Starch (mg·g^−1^)	55.494	0.000
NSCs (mg·g^−1^)	3.308	0.108
Soluble sugars/starch	10.27	0.012
Proline (μg·g^−1^ FW)	2.746	0.142
Soluble protein (mg·g^−1^ FW)	1.769	0.249
CAT (μmol·g^−1^ FW)	1.155	0.376
POD U·g^−1^·min^−1^ FW	6.756	0.029
SOD (U·g^−1^ FW)	13.475	0.006
MDA (μmol·g^−1^ FW)	1.18	0.370

Note: *F* indicates the effect of different numbers of drought–rehydration cycles on each indicator, *p* > 0.05 indicates no significant effect, *p* < 0.05 indicates significant effects, and *p* < 0.01 indicates highly significant effects.

## Data Availability

Data will be made available on request.
